# Awareness of Postdural Puncture Headache Among Specialists who Perform Lumbar Punctures and/or Monitor Patients Following the Procedure

**DOI:** 10.4274/TJAR.2023.221128

**Published:** 2023-06-16

**Authors:** Mesut Bakır, Şebnem Rumeli, Ümit Durmuşoğlu, Erman Balıkçı

**Affiliations:** 1 Clinic of Anaesthesiology and Reanimation, Mersin City Training and Research Hospital, Division of Algology, Mersin, Turkey; 2 Department of Anaesthesiology and Reanimation, Mersin University Faculty of Medicine, Division of Algology, Mersin, Turkey; 3 Department of Anaesthesiology and Reanimation, Mersin University Faculty of Medicine, Mersin, Turkey

**Keywords:** Adult anaesthesia, education and training, pain management, postdural puncture headache, regional anaesthesia

## Abstract

**Objective:**

Lumbar puncture (LP) is performed by specialists in different branches of medicine, complications may be encountered in various settings. In our study, we evaluated the awareness and knowledge of the diagnosis and treatment of post-dural puncture headache (PDPH) among specialists who performed LP and/or encountered complications.

**Methods:**

This was a prospective questionnaire/scale study of 253 physicians: LP performers (anaesthesiologists, Group A; others, Group B) and those who worked in departments that did not perform LP but frequently encountered complications following LP (Group C). The questionnaire assessed specialization, frequency of LPs utilization, needle types used, positions employed, awareness of LP complications, diagnosis, management, and risk factors for PDPH.

**Results:**

Group A had the highest percentage of physicians who stated they had knowledge about PDPH (Group A: 96.4%, Group B: 77.3%, Group C: 39.4%; P=0.000). Group C was found to be statistically less informed than the other two groups (*P*=0.000). It was determined that only one (1%) physician from Group C correctly answered the question about the diagnostic criteria for PDPH.

**Conclusion:**

To our knowledge this is the first study in which the awareness of PDPH has been compared according to physicians’ fields of specialisation. We believe that post-specialty training programs should be organized for physicians who will either perform LP or monitor patients who have undergone LP, and the curriculum content in relevant specialties should be reviewed.

**Main Points**

• The most frequent complication of lumbar puncture (LP) used for diagnosis or therapy is post-dural puncture headache (PDPH), which has a higher risk of morbidity and mortality if left untreated.

• If physicians recognize and treat PDPH at an early stage, this will significantly reduce the development of morbidity associated with this complication.

• Post-specialty training programs should be organized to better train physicians who will either perform LP or monitor patients who have undergone LP.

## Introduction

Postdural puncture headache (PDPH) is one of the most common complications of lumbar puncture (LP).^1^ Early diagnosis and treatment of PDPH are essential to prevent significant morbidity and mortality.^2^

LP is often performed by anaesthesiologists, paediatricians, neurologists, infectious disease, and emergency medicine specialists. Among other groups of physicians who must be aware of PDPH, one of the complications of the procedure, are physicians who do not perform this procedure but who frequently encounter these patients and are responsible for their follow-up and treatment, for instance, surgical department specialists. It has been reported in the literature that if anaesthesiologists and other physicians recognize and treat PDPH at an early stage, this will significantly reduce the development of morbidity associated with this complication.^3,4^

The factors that increase the risk of developing PDPH following LP are well defined in the literature. Among the patient groups at a high risk of developing PDPH are young people and women, particularly those who are pregnant.^5,6^ The needle size and type are also two important factors that increase the risk.^7,8^ The American Academy of Neurology recommends the use of small-scale atraumatic needles and placing the stylet into the needle in retries to reduce the risk of PDPH.^9^ Physicians should be fully aware of these factors that increase the risk of PDPH before performing LP.

The symptoms of certain diseases with high mortality, such as meningitis, intracranial hemorrhage, and sinus vein thrombosis, may be similar to the symptoms of PDPH. Physicians should have sufficient knowledge to make a differential diagnosis in patients suspected of having PDPH.

In our study, we aimed to evaluate the awareness of PDPH and the knowledge of its diagnosis and treatment among specialists who applied LP and/or encountered LP complications.

## Methods

A prospective questionnaire/scale study was conducted between 01.03.2020 and 15.03.2020. With ethics committee approval (date: 19.02.20, issue no.: 2020/184), a total of 255 local physicians were enrolled, consisting of LP performers (anaesthesiologists, Group A; others, Group B) and those working in departments that do not perform LP but may frequently encounter patients experiencing LP complications (Group C; Table 1). Non-active physicians and general practitioners were not included in the study.

The questionnaire consisted of 17 open-ended and multiple-choice questions for which many had answers ordered within a specific system. The principles of objectivity and avoidance of leading the participant to certain answers were employed when setting the question options. Participants were not asked for private information such as their name or the institution they worked for; we merely collected basic demographic data using simple closed questions. An information letter for the participants on the purpose and nature of the questionnaire was given in its introduction. There was no time restriction for filling out the survey. The questionnaire covered covered topics such as the residency program attended, number of LPs performed, types of needles used, positions applied, awareness of LP complications, knowledge of PDPH diagnosis, treatment approaches, and risk factors.

Physicians were enrolled in the study by emailing them the questionnaire. Support was received from the national anaesthesia association and physician communication networks in the relevant specialties in our province to obtain email contact information for the physicians. Only two questionnaires with incomplete answers were excluded from the study, resulting in a total of 253 sets of data available for analysis.

When reviewing the curricula for specialty education in our country, we found that course contents on intervention, diagnosis, and treatment of PDPH were available for anaesthesiologists and specialty physicians performing LP (Table 2).

### Statistical Analysis

Data for statistical evaluation were entered into the Statistical Package for Social Sciences version 24 (SPSS v.24) program. The E-PICOS program was also used to make calculations in line with the MedicReS Good Biostatistical Practice principles. Descriptive statistics were employed for categorical variables, and frequency calculations were expressed in percentage terms. The chi-square test was applied for cross-comparison tables. Independent- and dependent-group *t*-tests were performed for comparison of the mean values. A *P* value <0.05 was considered statistically significant.

## Results

The study included a total of 253 physicians, with 110 (43.5%) in Group A, 110 (43.5%) in Group B, and 33 (13.0%) in Group C.

**Figure 1:**
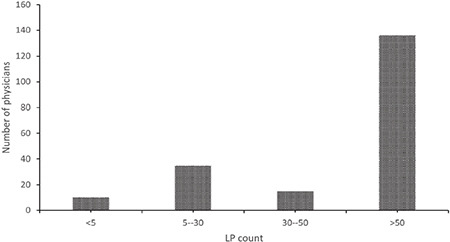
Number of LPs performed by physicians. LP, lumbar puncture.

Among the participants, 197 physicians reported performing LP (Group A, n = 104, 52.8%; Group B, n = 93, 47.2%). The rate of those with 50 or more LPs performed was found to be 53.8% (n = 136; Figure 1). More specifically, the rates were 86.4% (n = 95) in Group A and 37.3% (n = 41) in Group B. One physician did not specify the number of procedures performed.

The rates of physicians who stated they had knowledge about PDPH in the questionnaire were 96.4% in group A (n = 106), 77.3% in group B (n = 85), and 39.4% in group C (n = 13). It was determined that the awareness of group A was statistically higher than that of the other two groups (*P*=0.000). Group C was found to be statistically less informed compared to the other two groups (*P*=0.000). A total of 49 physicians (19.4%) stated that they did not have any information on PDPH.

**Figure 2:**
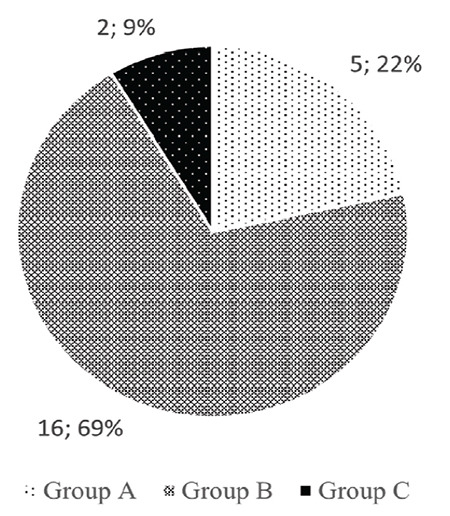
The ratio of those who did not encounter PDPH according to the physician groups. PDPH, post-dural puncture headache. n; %

Most (69.6%, n = 16) of the 23 physicians who had not encountered PDPH before were in Group B (p = 0.029; Figure 2). Among the other complications of lumbar puncture that were noted besides PDPH, 47.4% (n = 120) of the physicians reported having encountered lower back pain and 3.95% (n = 10) meningitis.

The physicians were found to use a 25-gauge (G) needle (n = 90, 45.7%) most frequently for LP. Almost all (95.2%, n = 40) of the 42 physicians using a 20-G needle were in group B (Table 3).

Of the 155 physicians who stated their needle type preferences, 74.2% (n = 115) preferred Quincke, 32.2% (n = 50) atraumatic, and 6.46% (n = 10) preferred both. More specifically, 75.5% (n = 83) of those in group A stated that they preferred Quincke and 26.4% (n = 29) atraumatic. The rate of those without any idea about the needle type was 21.3% (n = 42), and all of them were in group B.

Among those who stated that they used only the lateral position for patients during the LP procedure, 7.1% (n = 5) were in group A, while this rate was 92.9% (n = 66) in group B.

The rate of those who marked the three patient groups at a high risk of PDPH as pregnant, female, and young was 39.1% (n = 99). More specifically, 63.6% (n = 63) of those were in Group A, 30.3% (n = 30) in Group B, and 6.1% (n = 6) in Group C. Group A had a significantly better understanding of the risk groups compared to the other groups (*P*=0.000).

**Figure 3:**
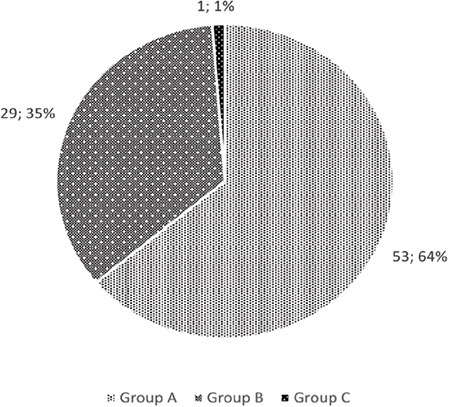
Distribution of those who correctly identified the diagnostic criteria by groups. n; %

Only one physician (1%) from Group C correctly listed the diagnostic criteria for PDPH determined by the International Headache Society (*P*=0.001; Figure 3).

**Figure 4:**
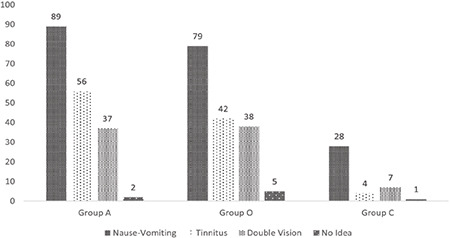
The distribution of those who knew the findings supporting the diagnosis of PDPH by groups. PDPH, post-dural puncture headache.

The only statistically significant difference between the groups was that fewer physicians in group C knew that tinnitus was a supporting criterion compared with the other groups (*P*=0.000) Figure 4.

The knowledge about drugs and methods used in PDPH treatment varied among the groups (Table 4), with epidural blood patch (EBP) being the least known method.

In the differential diagnosis, meningitis was the most considered disease by physicians in all groups (Table 5).

## Discussion

To our knowledge, this study is the first to compare the awareness levels of PDPH held by different specialist groups. Among the specialist physicians who performed LP or encountered patients experiencing LP complications, it was found that anaesthesiologists had greater awareness of the risk groups, diagnostic criteria, differential diagnosis, and treatment compared with other groups. However, anaesthesiologists preferred the seated position more than other specialists, although it is among the factors that increase the risk of PDPH. Meanwhile, although other specialist groups frequently performed LP with the patient positioned lying down, they preferred large needle diameters for the intervention, which also increase the risk of PDPH. In addition, it was found that physicians in all specialties had insufficient information about the supporting findings in the diagnosis of PDPH.

It has been reported that the incidence of PDPH varies between 6 and 36% after LP.^10^ In our study, the rate of physicians who stated that they had knowledge about PDPH was statistically significantly higher in group A compared with the other groups. We propose that the reason we found a difference in knowledge about PDPH was that anaesthesiologists performed LP more frequently after their residency compared to other groups and therefore were more likely to encounter PDPH at a time when they could apply learnings recently gained through specialty education, thus retaining such knowledge. Additionally, the knowledge of group C was significantly lower. It is obvious that the awareness of group C should be increased in their practice.

The literature contains few studies on the awareness of physicians regarding PDPH. In a survey conducted by Salzer et al.^11^, it was reported that only one in eight Swedish neurologists followed the algorithms recommended during LP to prevent PDPH, and two-thirds of the participants were found never to have used atraumatic needles before, a finding similar to that in our study concerning the use of atraumatic needles. Davis et al.^12^, meanwhile, showed that the use of atraumatic needles decreases the incidence of PDPH.

Many studies in the literature have stated that large-diameter needles are a risk factor. In one study, 22% of patients were found to develop PDPH with small- and 30.2% with large-diameter needles.^13^ In our study, it was found that physicians other than anaesthesiologists preferred large needle diameters. It is possible that these groups of physicians prefer a larger diameter because it enables them to collect more CSF, which supports them with the differential diagnosis of patients, or perhaps they can apply drugs more easily during intrathecal treatment with large needle diameters. As a next step, to refresh the knowledge of these physician groups on the relationship between needle diameter and PDPH, we believe that specialty education institutions should intervene to increase their awareness by creating tailored “recap” training materials.

In another area of research, previous studies investigated the patient position during LP and the development of PDPH. In one study, 125 patients who underwent LP were divided according to the procedure position into lateral and seated positions, and the incidence of PDPH was found to be significantly lower in lateral-position LPs.^14^ It was notable that most of the physicians who preferred the lateral position in our study were in group B. However, the same group chose a larger needle diameter, thus increasing the risk of the intervention, which poses a question whether they prefer the position in terms of low risk, ease of application or for another reason. We think that the real reason can be revealed through future studies.

While physicians’ experience increases as the number of procedures performed increases, their experience of encountering complications may not increase. In a study by Flaatten et al.^15^, clinicians were divided into five groups according to their degree of experience. The researchers then examined the PDPH complications patients developed in a follow-up of 100 LPs they performed, and no significant difference was found between the groups.^16^ In our study, it was found that the anaesthesiologists had both better knowledge of PDPH and a higher average count of LPs performed compared to the other groups. However, we cannon comment on whether their LP accordingly resulted in fewer complications because the PDPH frequency was not assessed in our study. Low awareness of PDPH in group C, where physicians never performed LP but monitored patients at risk of LP complications, suggests a serious knowledge deficiency for this group in terms of the condition diagnosis and treatment, which should be overcome through training programs organized by specialist associations.

It has been previously shown that young, female and pregnant patients are in the PDPH risk group.^17^ In a study by Khlebtovsky et al.^18^ conducted on 144 patients, PDPH risk factors were investigated and the risks for females and young people were found to be statistically significantly higher than those for other groups. In our study, the anaesthesiologists who had the highest knowledge about PDPH were the physicians who knew the risk groups best. Because group C included gynecologists and obstetricians, we thought we would find the best knowledge of high-risk groups among its physicians. However, the findings showed that the level of awareness was the lowest in this group, potentially because physicians in group C do not feel responsible for PDPH treatment.

Evaluation of additional symptoms in the differential diagnosis is important for early diagnosis. Ignoring additional symptoms may delay the diagnosis, result in extra interventional treatment steps, and increase the risk of morbidity. Turnbull et al.^19^ reported that symptoms such as nausea/vomiting, tinnitus, and double vision accompanies PDPH. Of the physicians participating in our study, 22.5% knew these were additional symptoms of PDPH, which indicates that physicians are not sufficiently aware of the additional criteria for the condition.

The most important diseases to be considered when making a differential diagnosis of PDPH are intracranial bleeding and meningitis. Meningitis was identified by 73% of the physicians in this study, while the identification rate of intracranial bleeding was 30%. In the future, we believe that both physicians who perform LP and those who monitor patients who have undergone LP should be made aware of intracranial haemorrhages, which may result in mortality if not diagnosed immediately and treated appropriately.

When non-invasive methods fail in PDPH treatment, the gold standard treatment is the epidural blood patch (EPB).^20^ Again, in this study, the majority (63.9%) of individuals who were unaware that EPB is included in the PDPH treatment algorithm belonged to Group B. This finding suggests that awareness that PDPH should be particularly increased in this group. A timely applied EPB prevents morbidity associated with PDPH, and therefore, we believe that its inclusion in the treatment algorithm should be commonly known among physicians.

### Study Limitations

The data included in the study were merely self-reported by those who participated in the survey. Furthermore, we surveyed only specialists in a single city, and we could not reach the sample size required to represent each specialty groups nationwide. In a further limitation, the core training objectives for specialization branches in our country have been shaped in the last 10 years; yet, in this study, no grouping was made according to the time when specialty education was completed. Therefore, unfortunately, any difference in knowledge between those educated before and after the curriculum restructuring could not be assessed.

## Conclusion

In conclusion, PDPH, the most common complication that follows LP performed for diagnosis or treatment, is a condition with increased morbidity and mortality risk when it goes untreated. In our study, although the rate of PDPH diagnosis was found to be quite high among the physicians who performed LP, the awareness about its differential diagnosis and treatment steps was found to be quite low. We propose that post-specialty training programs should be organized to better educate physicians who will either perform LP or monitor patients who have undergone LP, and the curriculum content in relevant specialties should be reviewed.

## Figures and Tables

**Table 1:**
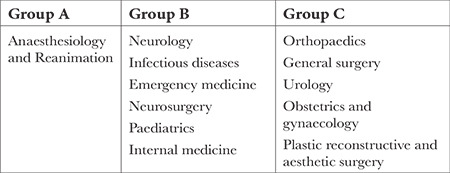
Groups of Physicians According to their Specialty

**Table 2:**
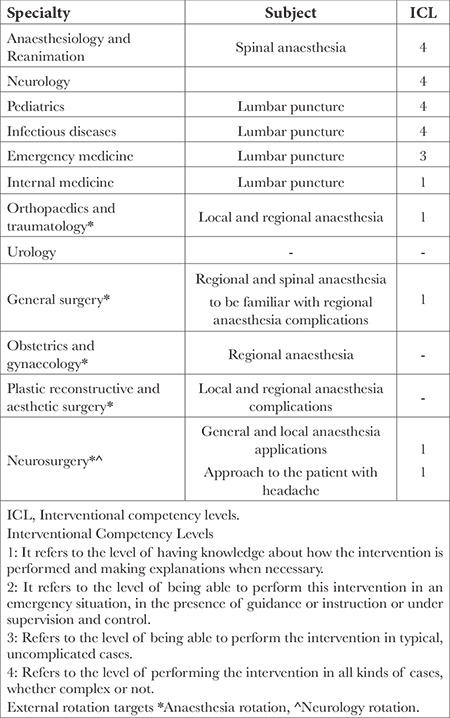
Subject headings and specified competency levels that may be associated with lumbar puncture attempts in the training curricula of the specialties

**Table 3:**
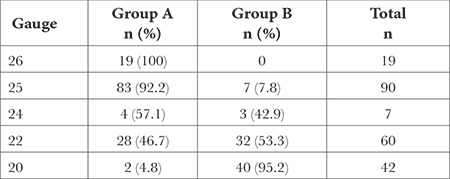
Distribution of Gauges of the Preferred Needles for Lumbar Puncture

**Table 4:**
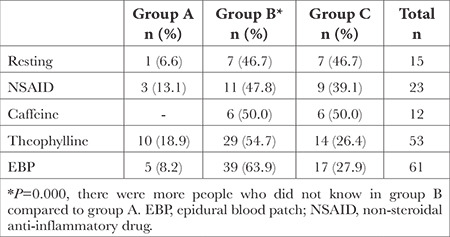
Distribution of Those Who Have No Idea About the Drugs and Methods Used in PDPH Treatments, by Groups

**Table 5:**
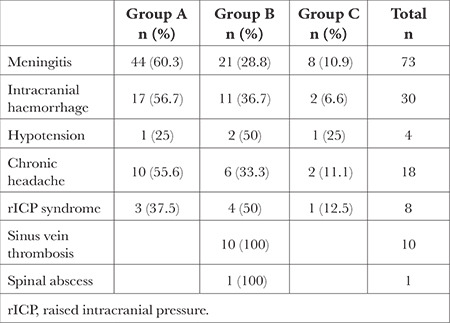
Distribution of Diseases Considered in Differential Diagnosis by Groups n (%)
